# OptiCogs: feasibility of a multicomponent intervention to rehabilitate people with cognitive impairment post-stroke

**DOI:** 10.1186/s40814-023-01300-7

**Published:** 2023-10-18

**Authors:** Mairéad O’ Donoghue, Pauline Boland, Sinead Taylor, Edel Hennessy, Eva Murphy, Siobhan Leahy, John McManus, Dominika Lisiecka, Helen Purtill, Rose Galvin, Sara Hayes

**Affiliations:** 1 School of Allied Health, Faculty of Education and Health Sciences, Ageing Research Centre, Health Research Institute, University of Limerick, Limerick, V94 T9PX Ireland; 2 Acute Stroke and Neurology Services, UL Hospitals Group, University Hospital Limerick, Limerick, Ireland; 3 Early Supported Discharge, UL Hospitals Group, University Hospital Limerick, Limerick, Ireland; 4 Department of Sport, Exercise and Nutrition, School of Science and Computing, Mayo Institute of Technology, Dublin Road, GalwayGalway, Ireland; 5 Department of Nursing and Healthcare Sciences, School of Health and Social Sciences, Munster Technological University Kerry Campus, Kerry, Ireland; 6 Department of Mathematics and Statistics, University of Limerick, Limerick, Ireland

**Keywords:** Stroke, Cognitive impairment, Multicomponent intervention, Feasibility

## Abstract

**Background:**

Stroke is a leading cause of death and disability worldwide. Despite the prevalence and associated burden of cognitive impairment post-stroke, there is uncertainty regarding optimal cognitive rehabilitation for people post-stroke. This study aimed to assess whether a multicomponent intervention, called OptiCogs, is feasible, acceptable, and safe for people with cognitive impairment post-stroke. A secondary aim was to explore changes in cognitive function, fatigue, quality of life, physical function, and occupational performance, from pre-intervention to post-intervention.

**Methods:**

A feasibility study was conducted where people post-stroke with cognitive impairment enrolled in a 6-week multicomponent intervention. The primary outcomes recorded included response rate, recruitment rate, retention rate, adherence to the intervention protocol, adverse events, and acceptability of the intervention to people post-stroke. Secondary outcomes included (i) change in cognitive functioning using the Addenbrooke’s Cognitive Examination III, (ii) fatigue using the Fatigue Severity scale, (iii) quality of life using the Stroke Specific Quality of Life scale (iv) physical function using the patient-reported outcomes measurement information system, and (v) patient-reported occupational performance using the Canadian Occupational Performance Measure. The Consolidated Standards of Reporting Trials extension reporting guidelines were followed, for pilot and feasibility studies, to standardize the conduct and reporting of this study.

**Results:**

The response rate was 10.9%. Nine eligible participants were enrolled during the 4-month recruitment period, with eight participants completing the entire 6-week intervention, as well as the pre- and post-intervention outcome measures. There were no reported adverse events. Participants were satisfied with the intervention and found it acceptable overall. Results of the secondary outcomes were promising for cognitive function (ACE III, pre: 63.3 ± 23.9 to post: 69 ± 24.6), fatigue (FSS, pre: 52.5 ± 7.3 to post: 45.6 ± 7.2), quality of life (SSQoL, pre: 131.0 ± 26.3 to post: 169.9 ± 15.3), physical function (PROMIS-PF, pre: 15.5 ± 6.3 to post: 15.8 ± 5.3), and occupational performance (COPM performance, pre: 9.3 ± 2.3 to post: 22.9 ± 4.2) and COPM satisfaction, pre: 9.9 ± 2.1 to post: 22.7 ± 3.5).

**Conclusion:**

Preliminary results suggest low-modest recruitment and high retention rates for the OptiCogs intervention. Changes in cognitive function, fatigue, quality of life, and self-reported occupational performance show improvement from pre- to post-intervention. These potential benefits require further testing in a larger pilot trial.

**Trial registration:**

NCT05414539.

**Supplementary Information:**

The online version contains supplementary material available at 10.1186/s40814-023-01300-7.

## Key messages regarding feasibility

1. What uncertainties existed regarding the feasibility?

Key uncertainties with regard to the recruitment, Retention, and adherence rates to a multicomponent intervention for PpS with cognitive impairment were addressed.

2) What are the key feasibility findings?

A combined cognitive-physical intervention of a 6-week duration is feasible to implement, Is largely acceptable to PpS, and is a safe intervention to implement for PpS with cognitive impairment

3) What are the implications of the feasibility findings for the design of the main study?

Components of the intervention schedule, Such as the optimal method of delivery of the intervention components, require modification prior to a future pilot RCT.

## Background

Stroke is among the leading causes of death and disability-adjusted life years worldwide [1]. Cognitive impairment in people post-stroke (PpS) occurs in up to 60% of people with ischemic stroke [2] with varying incidence rates between 20 and 80% of PpS [3–5]. Over 50% of PpS who recover well from the physical effects of stroke experience long-term cognitive deficits [6]. Data from a prospective population-based stroke register found the prevalence of cognitive deficits in 22% of people at 5 years post-stroke and 21% of people at 14 years post-stroke [7]. Cognitive impairment post-stroke is independently associated with a poorer quality of life [8], higher rates of mortality and institutionalization [9], increased caregiver burden [10], and increased healthcare costs [11].

A priority-setting partnership in the UK, the James Lind Alliance, identified that cognitive impairment post-stroke was the leading priority among the top 10 research priorities for PpS [12]. Despite this, much stroke rehabilitation focus is placed on the improvement of physical deficits, [13, 14], with PpS reporting a focus placed on physical needs over non-physical needs such as social re-integration and psychological support [15]. Previous Cochrane reviews have explored the effectiveness of cognitive rehabilitation interventions on single domains of cognitive function post-stroke, such as memory, executive function, attention, apraxia, neglect, and perception [16–21].

An overview by Gillespie et al. [22] synthesized evidence across these Cochrane reviews and reported favorable outcomes of cognitive rehabilitation across the domains of attention, spatial neglect, and motor apraxia immediately post-intervention. However, Gillespie et al*.* [22] noted that improvements were not likely to persist in the long-term and did not improve the function of PpS. Given the multi-faceted, and often diffuse nature, of cognitive impairment post-stroke, focusing on domain-specific cognitive outcomes post-stroke, may fail to capture the interconnected cognitive deficits which present in PpS [3, 23]. There remains a need to move beyond the narrow scope of specific cognitive rehabilitation interventions focusing on one specific domain of a cognitive function.

There appears a pressing need to develop a comprehensive and clinically relevant rehabilitation intervention for people with cognitive deficits post-stroke. The Medical Research Council (MRC) Framework offers guidance on the development and evaluation of complex interventions [24]. This framework emphasizes developmental and early piloting phases of interventions, wherein iterative and flexible processes are used to guide development. For instance, the first development phase of the MRC framework highlights the importance of identifying the existing evidence-based. O’Donoghue et al. (2022) conducted a systematic review and meta-analysis of 64 studies, addressing all types of non-pharmacological rehabilitation interventions which may improve multiple cognitive domains in PpS. Within this review, rehabilitation interventions were categorized as multicomponent interventions, physical activity interventions, cognitive rehabilitation interventions, non-invasive brain stimulation protocols, occupational-based interventions, and other interventions. The most consistent evidence in this review supported multicomponent interventions, with significant improvement demonstrated for general cognitive function (MD 1.56, 95% CI 0.69, 2.43) and memory (SMD 0.49, 95% CI 0.27, 0.72). Within these analyses of multicomponent interventions, cognitive rehabilitation training was used in conjunction with a form of physical activity. Physical activity interventions improved outcomes of neglect (MD 13.99, 95% CI 12.67, 15.32) and balance (MD 2.97, 95% CI 0.71, 5.23). Occupational-based intervention conducted within 3 months post-stroke showed an effect in favor of the intervention group for general cognitive function (MD 0.39, 95% CI (0.02 to 0.76).

Alongside this meta-analytic evidence, and in keeping with the MRC framework guidance of engaging key stakeholders [24], an in-depth qualitative study was conducted with PpS, caregivers, and healthcare professionals working in stroke rehabilitation [25]. Factors identified by stakeholders as being key to intervention development emphasized meaningful engagement, both in the type of intervention delivered, as well as the intervention setting [25]. The capacity of PpS to engage in rehabilitation, as well as the optimal timing and delivery of the intervention, was highlighted [25]. Drawing on this qualitative evidence, in conjunction with meta-analytic evidence from our systematic review, a multicomponent rehabilitation intervention aimed at improving cognitive functioning in PpS was systematically developed which we called Opticogs.

Randomized controlled trials are required to provide a robust evidence-based for a complex multicomponent intervention to improve cognitive functioning in PpS. The feasibility of such an intervention requires examination prior to proceeding with a pilot randomized controlled trial. The delivery approach of OptiCogs was created in response to public health guidelines aligned with the COVID-19 pandemic and thus adopted a telehealth approach to intervention delivery [26] and presents the proof of concept testing of OptiCogs in preparation for larger scale evaluations, in accordance with the MRC framework [24].

### Specific aims

The primary aim of this study was to assess the feasibility, acceptability, and safety of a multicomponent rehabilitation intervention for PpS with cognitive impairment. The secondary aim was to explore pre-to-post-test changes in outcome measures relating to cognitive function, fatigue, quality of life, physical function, and patient-reported occupational performance as proof of concept for a future larger-scale investigation.

## Material and methods

### Participants and setting

This study was conducted as a single-arm feasibility study using a pre-test to post-test design. The study design was chosen with the aim of testing the feasibility of a multicomponent rehabilitation intervention for people with cognitive impairment post-stroke [27]. The Consolidated Standards of Reporting Trials (CONSORT) extension for pilot and feasibility studies was followed to standardize the conduct and reporting of this study [28]. Ethical approval was obtained from the HSE-Mid Western Area Research Ethics Committee [REC: Ref: 121/2021] and the University of Limerick, Faculty of Education and Health Sciences Research Ethics Board [REC: 2022_03_07_EHS (OA)].

### Participant eligibility

#### Inclusion criteria

- People with a diagnosis of stroke. Stroke may have been ischaemic or hemorrhagic in nature.
- People aged ≥ 18 years old; with confirmed mild to moderate cognitive impairment using the Addenbroke’s examination III (ACE III) with a cutoff score of 88/100 [29].
- People post-stroke who had a modified rankin Scale (MRS) score of 0–3
- People post-stroke who were able to express their basic needs, verbally or not
- Had access to a smartphone, laptop, or tablet with an internet connection

#### Exclusion criteria

- Physician-confirmed contraindications for undertaking physical activity, e.g., safety, presence of unstable heart disease
- People with diagnosed TIA
- People post-stroke with known active delirium or dementia
- People post-stroke with a diagnosis of known pre-stroke cognitive impairment
- People post-stroke with moderate or severe visuospatial neglect

### Sample size

The target sample size of *n* = 10 participants was based on a pragmatic approach to the assessment of feasibility in the context of the study resources (i.e., funding and personnel) [30]. Recruitment took place over 12 weeks, where all eligible participants who expressed interest in participating in the study were invited to partake.

### Procedures

Participants were scheduled to complete baseline data collection during the pre-assessment week, i.e., week 0 of the intervention schedule (see Supplementary Table 1). Data collection took place in one-to-one sessions with the first author (MOD) via Microsoft Teams, and the ACE III measure was completed to establish participants’ pre-intervention cognitive function. A questionnaire was sent to all participants using MS forms, where participants were asked to complete demographic questions about their age, gender, education level, comorbidities, and rural/urban living environment. Data were collected the week subsequent to the completion of the 6-week intervention. These data were collected in the same sequence (one-to-one MS team meeting and questionnaires via MS forms).

### Patient and public partnership statement

A partnership-focused framework [31] guided the establishment of a patient and public partnership (PPI) panel of PpS and clinical experts in stroke rehabilitation to inform this study. In line with the INVOLVE principles and values [32], a PPI panel was established including three PpS with self-reported cognitive impairment, and three clinical experts in stroke rehabilitation, namely a Clinical Specialist Occupational Therapist in stroke rehabilitation, a senior Occupational Therapist and senior Physiotherapist in Early Supported Discharge for stroke. PPI began in November 2021, with panel members actively advising the research team on intervention refinement of OptiCogs until week 1 of intervention delivery which began in May 2022.

In terms of intervention refinement of OptiCogs, the PPI panel was initially consulted in November 2021, with the panel members giving their insights and MOD facilitating the sessions. A decision was made for PpS and clinical experts to meet separately in order to promote fairness of opportunity in line with the INVOLVE guidelines (Hayes et al. 2012). To this end, an agenda was set for each PPI meeting in advance, wherein the PPI panel members reviewed the meeting agenda and were encouraged to contact MOD should any queries arise. The agenda for the series of meetings with PpS and clinical experts in stroke rehabilitation were as follows:

**Table:** 

Meeting date and agenda with clinical experts	Meeting agenda	- Outcomes of meeting
Meeting 1 (November 2021): Intervention development	The preliminary intervention schedule was sent to the intervention development one week in advance of the meeting for their review	**-** Strengths and weaknesses of the intervention schedule were discussed **-** Approaches to facilitate optimal engagement in an online cognitive rehabilitation intervention post-stroke were discussed **-** MOD re-drafted the intervention schedule post-meeting and circulated it to panel members for any further feedback
Meeting 2 (February 2022): Recruitment and further review of intervention schedule	Given the slow recruitment rates from UHL, input was sought from clinical experts on how to expand recruitment channels The panel was asked to review the OptiCogs intervention program and outline any changes they would recommend to facilitate optimal engagement	**-** Recruited was extended to other clinical sites within the UL Hospitals group namely, St. Camillus and St. Ita’s Hospital Newcastle West, as well as recruiting via a gatekeeper from the Irish Heart Foundation **-** The intervention schedule was shortened from including 6 cognitive domains to 3 cognitive domains as follows: attention, memory, and executive function
Meeting date and agenda with people post-stroke with self-reported cognitive impairment	**Meeting agenda**	**Outcomes of meeting**
Meeting 1 (February 2022): Introduction to other panel members and the research topic	The panel members were introduced to one another The boundaries of the group were discussed as well as the optimal approach to conduct the meetings, i.e., it was decided that a meeting agenda be sent out one week in advance of the meeting in order for time to read the information and pose any queries to MOD prior to the meeting date	A safe and friendly environment was created where PpS felt empowered to express their opinions in line with the INVOLVE guidelines and fairness of opportunity to contribute
Meeting 2 (March 2022): Review of the participant information leaflet and discussion of the recruitment process	The panel was asked to review the participant information leaflet	Following their review, proposed changes to the leaflet from panel members were as follows: **-** Fewer words overall **-** Use of larger fonts **-** Visuals to convey the message quickly **-** Use of layman terms and less use of “research terms” **-** Additional information regarding the ability of participants to reach out for support during the intervention should any needs arise and know that their needs will be listened to, and dealt with accordingly and with confidentiality
Meeting 3 (April 2022): Proposed change of method of delivery of OptiCogs from face-to-face to an online method of intervention delivery	The panel was asked to detail their views on the potential to run OptiCogs via an online method of delivery in response to Covid-19	**-** The pros and cons of telehealth were discussed **-** The panel members emphasized that small group numbers were key, i.e., no more than 3 participants per Teams call

The main objective of the panel was to provide consensus on key aspects of the intervention design. This objective was achieved through collaborative interpretation of the existing evidence, in conjunction with patient values and clinical expertise. Feedback on the feasibility and acceptability of the intervention schedule was gathered from a series of meetings with stakeholders. Key principles of complex intervention development are that it is, “dynamic, iterative, creative, open to change and forward-looking to future evaluation and implementation” [33]. In accordance with these principles, the clinical experts in stroke rehabilitation detailed what would be feasible in clinical practice, given their experience in the context in which OptiCogs would be delivered. This input was balanced with the continual input from PpS, who provided their insights into intervention development drawing from their experiential expertise of stroke, gained from their lived experiences [34].

### Description of OptiCogs

OptiCogs is a 6-week multicomponent intervention, consisting of both cognitive rehabilitation and physical activity components. The cognitive component was delivered via telehealth by an occupational therapist, while the physical activity component was delivered via telehealth by a chartered physiotherapist and first author (MOD).

For the cognitive component of the intervention, there were both group-based cognitive intervention sessions, as well as three individualized one-to-one cognitive sessions for each participant. The individualized component was devised drawing on the goals of the PpS identified via the COPM pre-intervention. One-to-one cognitive sessions were tailored in accordance with the cognitive profile of the PpS, based on scores from the ACE-III as well as their established pre-assessment goals using the COPM [35]. For full details of the cognitive component of the intervention schedule, please see supplementary Table 1. Group-based domain-focused cognitive intervention was delivered in relation to attention, memory, and executive function.

The physical activity component was informed by exercise recommendations for stroke survivors [36] and supplemented by findings of our quantitative systematic review and qualitative findings [25, 37]. Group-based exercise classes, delivered via telehealth, adopted a circuit class style, with sessions progressively increasing in intensity from week 1 to week 6. Sessions included a full body warm-up for approximately 5 min, followed by a circuit of eight strengthening exercises, targeting each major muscle group. Sessions were structured to allow for progressive overload, wherein participants were educated on how to exercise to fatigue on each set of the resistance exercises. For full details of the physical activity component of the intervention schedule, please see Supplementary Table 1.

### Primary outcome measures

The recruitment response rate, expressed as a percentage, was computed as the number of people recruited to the study divided by the number who were screened as eligible and invited to participate. The recruitment rate was calculated as a percentage, with the number of people enrolled (numerator) over the number of eligible participants (denominator). Retention was calculated as the proportion of individuals who, after enrolment, completed the pre-intervention outcome measures and post-intervention outcome measures. Adherence to the intervention schedule was assessed through recording attendance at all sessions. Reasons for non-attendance were recorded by facilitators. Participants were consulted by MOD on a weekly basis to document adherence to the intervention protocol, discuss if any adverse events were occurring or at risk, and any other issues raised by participants throughout the intervention period. Adverse events were assessed using a self-report diary and included (1) pain during or after a session, (2) fall during or after a session, (3) emotional distress, (4) exacerbation of fatigue, or (5) other neurological symptoms post-stroke. These were self-reported by participants in response to questioning by the facilitators (MOD) at the beginning of each session. Acceptability of the OptiCogs intervention was assessed using a self-report questionnaire developed by the researchers containing 5-point Likert scales: 1 = “strongly disagree,” 2 = “disagree,” 3 = “neutral,” 4 = “agree,” and 5 = “strongly agree.”

### Secondary outcome measures

The secondary objectives of this study were to examine changes in the following outcome measures following the OptiCogs intervention:

- Cognitive function: The Addenbrooke’s Cognitive Examination-III (ACE-III) is a cognitive test that assesses five cognitive domains namely, attention, memory, verbal fluency, language, and visuospatial abilities [38]. The optimal ACE-III cutoff scores to detect mild cognitive impairment are 88/100 (sensitivity 0.77, specificity 0.92) [29].
- Fatigue: The Fatigue Severity Scale (FSS) is a 9-item scale which measures the severity of fatigue and its effect on activities of daily living on a 7-point scale. A total score of 36 or more indicates that one is experiencing fatigue. The FSS is shown as a valid and reliable measure of fatigue in PpS [39].
- Quality of life: The Stroke-Specific Quality of Life Scale-12 (SSQoL-12) is a 12-item scale containing 49 items in 12 domains, namely, mobility, energy, upper extremity function, work/productivity, mood, self-care, social roles, family roles, vision, language, thinking, and personality. Each item is scored on a 5-point Likert scale with higher scores indicating better functioning [40]. The SSQoL-12 displays good criterion validity for all subsets of stroke [41].
- Physical function: The patient-reported outcome measurement information system (PROMIS) scale (PROMIS-10) scale is indicated for use by an international expert panel within the “standard set” of stroke measures [42]. The PROMIS-10 has been validated within the stroke population [43]. The physical function (PF) subset of the PROMIS-10 is shown to be feasible to obtain a measurement of PF in ischaemic stroke patients, with lower patient burden in comparison to the Stroke Impact Scale and minimal ceiling effect [44]. The items of the PROMIS-PF are scored numerically for an individual’s response to each question, with scores added up to find the total raw score.
- Participant-rated goal attainment: The Canadian Occupational Performance Measure (COPM) is a client-centred tool designed to capture the individual’s self-perception of performance within three occupational performance areas: self-care, productivity, and leisure [35]. The individual is asked to use a 10-point scale to rate their own level of performance and satisfaction with performance for each the five identified areas for improvement. The therapist calculates an average COPM performance score and satisfaction score, typically ranging between 1 and 10, with 1 indicating poor performance and 10 indicating very good performance and high satisfaction. Systematic review evidence shows the COPM to be an appropriate tool for clinicians in assessing outcomes and collaborative goal-setting in PpS, with good test–retest reliability within the stroke population [45].

### Statistical analysis

Summary statistics were used to describe participant characteristics at baseline using proportions (percentage) or means and standard deviations (SD). Numeric data were assessed for skewness using visual plots and the Shapiro–Wilk test of normality. Primary feasibility and adherence outcomes are reported as proportions. Analysis of the secondary outcomes variables compared differences between the pre-intervention and post-intervention scores using mean difference (post–pre) with associated 95% confidence intervals. SPSS software (Version 28.0 Armonk, NT: IBM Corp) was used for all analyses.

## Results

### Primary outcomes

#### Response rate

**Fig. 1:**
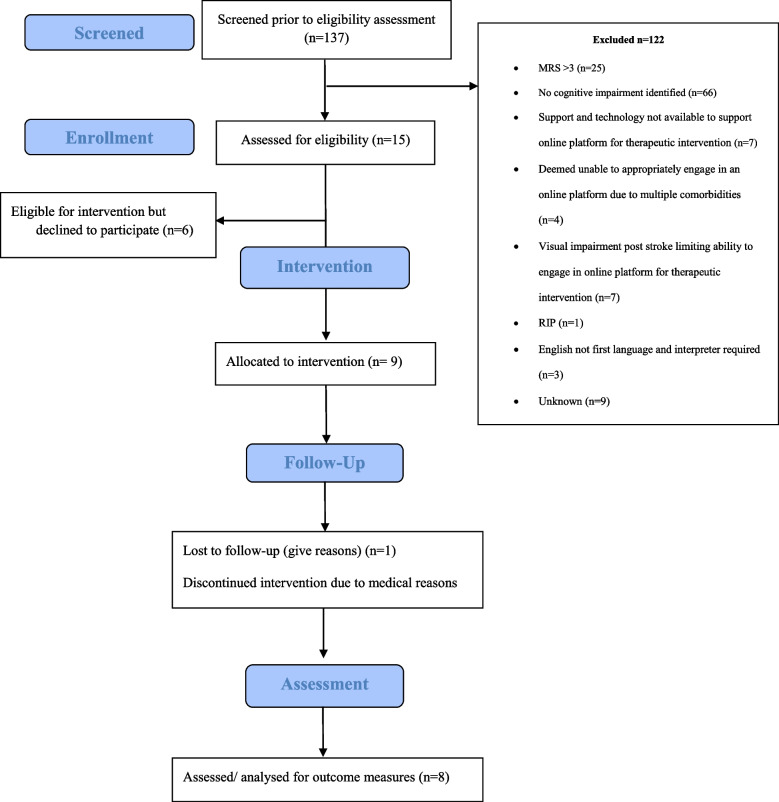
Participant flow diagram

As displayed in Fig. 1, the response rate was as follows: 15/137 × 100% = 10.9%.

#### Recruitment and retention rates

As displayed in Fig. 1, from February to May 2022, nine participants enrolled in the intervention, with eight participants completing the study. The target sample size of ten participants was not reached as a result of this screening process with eligibility criteria as outlined previously. Table 1 provides an overview of the baseline characteristics of participants. One individual withdrew from the study before commencing the intervention phase due to medical reasons. Therefore, eight participants in total (88.9%) completed the 6-week intervention and all outcome measures. Five participants attended all the physical and cognitive elements of the intervention. The flow of participants is outlined in Fig. 1.

**Table 1:** Participant demographics

**Gender**	
Female	4 (50%)
Male	4 (50%)
**Age**	55.8 (16.1)
**Marital status**	
Living with a partner	3 (37.5%)
Married	5 (62.5%)
**Employment**	
Retired	3 (37.5%)
Unable to work due to health reasons	1 (12.5%)
Work full-time	3 (37.5%)
Work part-time	1 (12.5%)
**Time post-stroke (years)**	2.2 (1.6)
**Type of stroke**	
Hemorrhagic stroke	2 (25%)
Ischemic stroke	6 (75%)
**Education level**	
Secondary school—leaving certificate	3 (37.5%)
Third level—diploma/certificate/degree	5 (62.5%)
**Mobility level**	
Ambulatory with bilateral assistance (canes, crutches, walker frame)	1 (12.5%)
Ambulatory without aid	7 (87.5%)
**Co-morbidities**	
Mild asthma	1 (12.5%)
Overweight	1 (12.5%)
Pacemaker	1 (12.5%)
**Previous fall**	
No	6 (75%)
Yes	2 (25%)

#### Adherence

The intervention consisted of six group-based cognitive rehabilitation sessions, six group-based physical activity sessions, and three one-to-one cognitive sessions. Six (75%) participants attended all three one-to-one cognitive sessions. Five participants (62.5%) attended all six group-based cognitive sessions. The group-based physical activity sessions were attended in full by four (50%) participants. Barriers such as difficulty in using the required hardware/software to attend telehealth sessions were recorded. Participants also reported challenges with monitoring emails for information on when to attend sessions. Also, the lack of a face-to-face meeting with facilitators and other participants was reported as the reasons for non-adherence by some participants.

#### Adverse events

There were no reported adverse events by participants during the intervention phase. One participant dropped out for a medical reason, which was independent of the intervention itself.

#### Acceptability

The responses to the participant acceptability questionnaire (Likert scale) are shown in Table 2. Mean scores ranged from 3.6 (SD ± 0.74) regarding the physical activity component of the intervention to 4.9 (SD ± 0.35) on the overall level of satisfaction with OptiCogs.

**Table 2:** Participant acceptability questionnaire

**Questions**	**Mean (SD)**
**Q1. I had good support from my healthcare professional during participation in OptiCogs**	4.8 (0.46)
**Q2. The content of the cognitive rehabilitation was acceptable and met my needs**	4.1 (0.64)
**Q3. The content of the physical activity component was acceptable and met my needs**	3.6 (0.74)
**Q4. The individualized calls with the occupational therapist were acceptable and met my needs**	4.3 (0.46)
**Q5. Taking part in OptiCogs for 6 weeks was acceptable and met my needs**	4.5 (0.53)
**Q6. Please, tick the appropriate box that indicates your overall satisfaction with OptiCogs**	4.9 (0.35)

5-point Likert scale employed across questions (score of 1 = “strongly disagree”; 2 = “disagree”; 3 = “neutral”; 4 = “agree”; 5 = “strongly agree

### Secondary outcomes

Secondary outcomes for pre- and post-intervention time points are provided in Table 3. Cognitive function as measured by the ACE III scores improved, MD = 5.8 (95% CI 0.9, 10.6). Fatigue severity as measured by the FSS decreased, MD =  − 6.9 (95% CI − 12.6, − 1.2). Changes in the quality of life post-stroke as measured by the SSQoL scale improved, MD = 38.9 (95% CI 19.7, 58.0). There was little change in physical function as measured by the PROMIS-10, MD = 0.3 (95% CI − 2.1, 2.6). COPM scores improved for performance, MD = 13.6 (95% CI 8.9, 18.3) and satisfaction, MD = 12.9 (95% CI 8.6, 17.1).

**Table 3:** Secondary outcomes pre- and post-intervention

	**Pre**	**Post**	**Mean difference (95% CI)**
ACE-III	63.3 (23.9)	69 (24.6)	5.8 (0.9, 10.6)
FSS	52.5 (7.3)	45.6 (7.2)	− 6.9 (− 12.6, − 1.2)
Global Fatigue	8.0 (1.4)	6.9 (1.4)	− 1.1 (− 1.8, − 0.4)
SSQoL	131.0 (26.3)	169.9 (15.3)	38.9 (19.7, 58.0)
PROMIS-PF	15.5 (6.3)	15.8 (5.3)	0.3 (− 2.1, 2.6)
COPM Performance*	9.3 (2.3)	22.9 (4.2)	13.6 (8.9, 18.3)
COPM Satisfaction*	9.9 (2.1)	22.7 (3.5)	12.9 (8.6, 17.1)

*ACE III *Addenbrooke’s Cognitive Examination III (higher scores reflect improvement in cognitive function), *FSS *Fatigue Severity Scale (lower scores reflect a decrease in fatigue), Global Fatigue scale (a subset of the FSS, wherein lower score reflects a decrease in fatigue),* PROMIS-PF *patient reported outcomes measurement information system-physical function subset,* SSQoL* Stroke Specific Quality of Life scale (higher score reflects an increase in quality of life), *COPM* Canadian Occupational Performance Measure (scores on level of performance and satisfaction of performance range from 1 to 10, where 1 indicates poor performance and low satisfaction, with 10 representing high performance and high satisfaction),*n = 7 participants completed the COPM measure

## Discussion

The current study findings demonstrated the feasibility, acceptability, and safety of this multicomponent intervention for people with cognitive impairment post-stroke. These findings will add to the future development of the intervention, wherein further development and refinement will be conducted in accordance with the MRC framework [24]. Participant behavior and feedback offer practical applications and clinical recommendations for future work in this field.

Participants reported high levels of satisfaction with the intervention schedule, with overall satisfaction with the intervention reported at 4.9 points (SD ± 0.35). Adherence rates, ranging from 50 to 75%, demonstrated modest rates of adherence for completion of the 6-week intervention phase of OptiCogs. Also, no adverse events were reported in this study. This study did not seek to examine if participants experienced significant changes in secondary outcomes from pre-intervention to post-intervention. However, there were improvements in secondary outcome measures relating to cognitive function, fatigue, quality of life, physical function, and goal attainment in occupational performance.

In terms of recruitment, retention, and adherence, the results of this study suggest some modifications to the intervention are necessary. The delivery of OptiCogs via telehealth, for example, resulted in barriers to recruitment and optimal engagement in the intervention for some. The delivery of OptiCogs was developed within the context of the COVID-19 pandemic and in keeping with public health guidelines adopted a telehealth approach to intervention delivery (World Health Organization 2021). In light of the barriers observed by participants engaging in OptiCogs, offering cognitive telerehabilitation post-stroke as the sole service model is not recommended and a more flexible model should be considered in the future including face-to-face, hybrid, and telehealth options. This mirrors current research into telehealth and stroke rehabilitation where the so-called digital divide results in participants encountering challenges in accessing digital technologies due to a lack of skills and education in technological usage [46].

We noted higher rates of adherence for the one-to-one therapy sessions. This may have been positively influenced by one-to-one contact with a healthcare professional during these individualized sessions. It is known that HCP communication is significantly positively correlated with patient adherence, wherein the odds of patient adherence are 1.62 times higher when a HCP is trained in communication skills [47]. This again suggests the importance of clinician-patient contact and points to the need to consider offering personalized and flexible choices to people with post-stroke cognitive impairment to optimize participation in such interventions. This individualized approach is supported by the work of Clare et al*.* [48] who conducted the “Goal-oriented cognitive rehabilitation for early-stage Alzheimer’s and related dementias: the GREAT RCT”. The GREAT RCT offered a cognitive rehabilitation program to *n* = 475 people with Alzheimer’s disease or vascular or mixed dementia or had mild to moderate cognitive impairment**.** GREAT was delivered by nine occupational therapists and one nurse, over a 3-month period in the participants’ homes. At three months, there were statistically significant large positive effects for participant-rated goal attainment as measured by the COPM [mean change in the CR arm: 2.57; mean change in the TAU arm: 0.86; Cohen’s *d* = 0.97, 95% confidence interval (CI) 0.75 to 1.19]. These positive findings for participant-rated goal attainment in the GREAT RCT are mirrored in the current study, wherein COPM scores improved for levels of satisfaction relating to goal attainment (MD = 12.9 (95% CI 8.6, 17.1) post-intervention, i.e., 6 weeks.

The results of this study provide preliminary evidence that a multicomponent intervention may be beneficial in improving cognitive function, fatigue, quality of life, and occupational performance in PpS. These potential benefits are tentatively interpreted and require further testing in a larger pilot randomized trial. In particular, changes in ACE III and SSQoL scores suggest improvements of cognitive function and quality of life post-intervention, i.e., 6 weeks. This mirrors previous literature wherein a combined intervention of physical activity and cognitive training significantly improved cognitive function [49–52] and quality of life post-intervention in people post-stroke [51].

The current study findings demonstrated a positive trend in reducing fatigue severity post-intervention. Also, there was a small trend toward improvement in physical function demonstrated post-intervention. These results are supported in a randomized controlled trial of *n* = 73 PpS who were randomized to either an intervention combining cognitive therapy with graded activity training (COGRAT) or cognitive therapy alone [53]. While this study found that both treatments demonstrated significant improvements on fatigue and physical endurance, the combined COGRAT intervention exceeded those of the single-component cognitive therapy intervention. Specifically, the number of participants showing clinical improvements in measures of fatigue was higher in the COGRAT versus the single component cognitive therapy (58% of participants versus 24% of participants) [53]. In addition, gains in the COGRAT group for physical endurance exceeded those of the single-component cognitive therapy intervention (*p* < 0.001) [53]. However, the low satisfaction rate in relation to the online delivery of the physical activity component must be noted. Participants encountered challenges with the completion of physical activity via telehealth, namely, difficulty following commands in the context of a group-based setting and difficulty with Internet connection. The current study suggests that telerehabilitation is likely most suitably situated as a component within a complex intervention, as opposed to being the sole method of intervention delivery. Its centrality to the delivery of the intervention may negatively impact the level of engagement from participants, a finding mirrored in the literature and the so-called digital divide referring to the lack of access to digital technologies and connectivity within certain populations, and the lack of motivation to engage in rehabilitation interventions delivered via telehealth [46]. PpS who experience communication and/or cognitive impairment are also likely to encounter more difficulty with telerehabilitation than those without [54] It seems therefore that offering telerehabilitation as the primary service model may not be recommended.

The results of this study must be interpreted in the context of its strengths and limitations. OptiCogs was developed following a comprehensive systematic review and meta-analysis [37], followed by an in-depth qualitative exploration of key stakeholders in stroke rehabilitation [25] and meaningful PPI engagement, guided by the MRC framework [24]. The transparent development of OptiCogs will allow for intervention replication and sets the foundation for a pilot RCT to be conducted, wherein estimate effect sizes will be calculated and further intervention refinement can be conducted in line with the MRC framework [24].

However, these preliminary data are encouraging and suggest a larger pilot study is warranted. In discussing the implications of the current study findings, the single-arm feasibility study design must be considered. Without a control group, it is difficult to ascertain how much of these improvements are as a result of natural recovery. Therefore, the potential benefits observed are interpreted with caution and require further testing in a larger pilot trial. Given the lack of blinded outcome assessment, the potential for detection bias is acknowledged.

With regard to the future intervention development of OptiCogs, the value of a process evaluation of OptiCogs should be considered. This process evaluation would further outline the potential modifications needed for OptiCogs. The MRC guidance on process evaluations described by Moore et al. (2015) suggests that early evaluation of these testing phases should encompass a process evaluation in which quantitative data is combined with an in-depth qualitative exploration to provide detail on the implementation and functioning of the intervention on a small scale [55]. Process evaluations serve a very important role in health service research by providing detailed insight into the experiences of those exposed to the intervention [56]. This is of particular note in stroke rehabilitation research where the translation of interventions into practice has been described as “impossible’, due to the prevalence of insufficient implementation strategies and methodological descriptions [57]. Trials which include a process evaluation are known to yield higher-quality results, which can help translate intervention findings and enhance the potential generalisability and optimization of the proposed intervention in clinical practice [58]. In this way, a process evaluation of OptiCogs would shed light on the previously described ‘black box’ of cognitive rehabilitation interventions [59]. Through the conduct of a process evaluation, OptiCogs could be evaluated further in terms of economic considerations, cost–benefit analysis, and the use of clinical outcomes to inform sample size calculation. To this end, future process evaluations of OptiCogs should be considered in line with the key recommendations regarding the planning, design, evaluation, and reporting of process evaluations for complex interventions [55].

Furthermore, the small sample size which limits generalisability, and so, these results should be interpreted with caution. On reflection, perhaps a more pragmatic approach to recruitment should have been taken. For example, alternative pathways of recruitment may need to be considered. Given the challenges to recruitment for rehabilitation studies in the early phase post-stroke [60], it is unsurprising that community-based stroke trials have the most efficient recruitment rates [61]. This is evident from the initial recruitment phase which via an acute hospital (the ESD stroke service in University Hospital Limerick) which resulted in one participant being recruited over a period of 4 months. Subsequently, having opened our recruitment to community-based stroke rehabilitation services via the Irish Heart Foundation, seven participants were recruited within one month. Thus, in consideration of the recruitment rates of this feasibility study, and in the context of the wider literature on stroke rehabilitation trials, it may be interpreted that PpS in the sub-acute to the chronic phase of recovery are in a better position to be approached regarding trial participation. Perhaps the immediate psychological and physical implications of stroke have subsided, and the most intensive period of standard rehabilitation is likely to have been carried out once in the sub-acute to chronic phase post-stroke. Furthermore, a lack of community care and limited support from primary healthcare services is known to contribute to a perception of marginalization and a feeling of abandonment by PpS and their caregivers following discharge from the hospital setting [62]. Again, this was mirrored by the qualitative findings of O’ Donoghue et al. [25] wherein PpS and carers felt isolated once discharged from acute stroke services and were back into the community. To this end, recruitment forecasting for a future pilot trial of OptiCogs should carefully consider the stage of rehabilitation post-stroke when specifying eligibility criteria and locations of recruitment.

Also, the delivery of OptiCogs was driven by public health guidelines aligned with the COVID-19 pandemic and thus adopted a telehealth approach to intervention delivery. However, there are barriers to telerehabilitation interventions and the so-called digital divide wherein participants encounter challenges in accessing digital technologies due to a lack of skills and education in technological usage [46]. There are complex challenges in embedding telerehabilitation services for PpS. A recent systematic review of 41 RCTs of stroke telerehabilitation interventions synthesized the barriers and facilitators of stroke telerehabilitation delivery [63]. While usability and acceptability with telerehabilitation were high across the included studies, issues relating to inaccessibility and technical challenges, as well as training requirements for intervention delivery were described as barriers to the adoption of telerehabilitation in stroke services [63]. These findings were mirrored in the current study, wherein PpS reported technical issues and the lack of clinician-patient rapport as barriers to participation in OptiCogs. It seems, therefore, that offering telerehabilitation appears feasible; however, modifications to OptiCogs are needed to ensure optimal engagement from PpS. To this end, the delivery of cognitive rehabilitation via telerehabilitation platforms as the primary service model may not be recommended and future research in this area is needed.

## Conclusions

To the authors’ knowledge, there are no studies investigating the effectiveness of a combined cognitive rehabilitation and physical activity multicomponent intervention for cognitive rehabilitation for PpS. While the results of this single-arm feasibility study indicate that some modifications to OptiCogs are needed prior to the commencement of a larger pilot trial, preliminary results suggest that OptiCogs is feasible, safe, and acceptable to PpS. Changes in cognitive functioning scores between baseline and post-intervention assessment suggest improvements in general cognitive functioning, fatigue, and quality of life outcomes. These findings serve as a preliminary step to inform the development of an evidence-based future pilot randomized control trial.

### Supplementary Information

**Additional file 1:** **Table S1.** Intervention schedule (OptiCogs).

## Data Availability

The datasets supporting the conclusions of this article are included within the article and its additional files.

## Abbreviations

ACE III: Addenbrooke’s Cognitive Examination III
CONSORT: Consolidated Standards of Reporting Trials
COPM: Canadian Occupational Performance Measure
FSS: Fatigue Severity Scale
MD: Mean difference
MRC: Medical Research Council
MRS: Modified Rankin Scale
PROMIS-PF: Patient-Reported Outcomes Measurement Information System-Physical Function subset
PPI: Patient and public involvement
PpS: Person post-stroke
SMD: Standardized mean difference
SSQoL: Stroke Specific Quality of Life Scale

## References

[1] Feigin VL, Norrving B, Mensah GA (2017). Global burden of stroke. Circ Res.

[2] Mellon L, Brewer L, Hall P, Horgan F, Williams D, Hickey A (2015). Cognitive impairment six months after ischaemic stroke: a profile from the ASPIRE-S study. BMC Neurol.

[3] Tatemichi T, Paik M, Bagiella E, Desmond D, Pirro M, Hanzawa L (1994). Dementia after stroke is a predictor of long-term survival. Stroke.

[4] Rasquin SM, Lodder J, Ponds RW, Winkens I, Jolles J, Verhey FR (2004). Cognitive functioning after stroke: a one-year follow-up study. Dement Geriatr Cogn Disord.

[5] Nys G, Van Zandvoort M, De Kort P, Jansen B, Kappelle L, De Haan E (2005). Restrictions of the Mini-Mental State Examination in acute stroke. Arch Clin Neuropsychol.

[6] Kapoor A, Lanctôt KL, Bayley M, Kiss A, Herrmann N, Murray BJ (2017). “Good outcome” isn’t good enough: cognitive impairment, depressive symptoms, and social restrictions in physically recovered stroke patients. Stroke.

[7] Douiri A, Rudd AG, Wolfe CD (2013). Prevalence of poststroke cognitive impairment: South London stroke register 1995–2010. Stroke.

[8] Cumming TB, Brodtmann A, Darby D, Bernhardt J (2014). The importance of cognition to quality of life after stroke. J Psychosom Res.

[9] Patel MD, Coshall C, Rudd AG, Wolfe CDA (2002). Cognitive impairment after stroke: clinical determinants and its associations with long-term stroke outcomes. J Am Geriatr Soc.

[10] Atteih S, Mellon L, Hall P, Brewer L, Horgan F, Williams D (2015). Implications of stroke for caregiver outcomes: findings from the ASPIRE-S study. Int J Stroke.

[11] Claesson L, Lindén T, Skoog I, Blomstrand C (2005). Cognitive impairment after stroke–impact on activities of daily living and costs of care for elderly people. Cerebrovasc Dis.

[12] Pollock A, St George B, Fenton M, Firkins L (2014). Top 10 research priorities relating to life after stroke–consensus from stroke survivors, caregivers, and health professionals. Int J Stroke.

[13] Hochstenbach JB, den Otter R, Mulder TW (2003). Cognitive recovery after stroke: a 2-year follow-up. Arch Phys Med Rehabil.

[14] Jacova C, Pearce LA, Costello R, McClure LA, Holliday SL, Hart RG (2012). Cognitive impairment in lacunar strokes: the SPS3 trial. Ann Neurol.

[15] Peoples H, Satink T, Steultjens E (2011). Stroke survivors’ experiences of rehabilitation: a systematic review of qualitative studies. Scand J Occup Ther.

[16] Lincoln N, Majid M, Weyman N. Cognitive rehabilitation for attention deficits following stroke. Cochrane Database Syst Rev. 2000;(4). 10.1002/14651858.CD002842 11034773

[17] das Nair R, Lincoln N. Cognitive rehabilitation for memory deficits following stroke. Cochrane Database Syst Rev. 2007;(3). 10.1002/14651858.CD002293.pub2 17636703

[18] West C, Bowen A, Hesketh A, Vail A. Interventions for motor apraxia following stroke. Cochrane Database Syst Rev. 2008;(1). 10.1002/14651858.CD004132.pub2 PMC6464830 18254038

[19] Bowen A, Knapp P, Gillespie D, Nicolson DJ, Vail A (2011). Non-pharmacological interventions for perceptual disorders following stroke and other adult-acquired, non-progressive brain injury. Cochrane Database Syst Rev.

[20] Bowen A, Hazelton C, Pollock A, Lincoln NB (2013). Cognitive rehabilitation for spatial neglect following stroke. Cochrane Database Syst Rev.

[21] Chung CS, Pollock A, Campbell T, Durward BR, Hagen S (2013). Cognitive rehabilitation for executive dysfunction in adults with stroke or other adult non-progressive acquired brain damage. Cochrane Database Syst Rev.

[22] Gillespie DC, Bowen A, Chung CS, Cockburn J, Knapp P, Pollock A (2015). Rehabilitation for post-stroke cognitive impairment: an overview of recommendations arising from systematic reviews of current evidence. Clin Rehabil.

[23] Ramsey L, Siegel J, Lang C, Strube M, Shulman G, Corbetta M (2017). Behavioural clusters and predictors of performance during recovery from stroke. Nat Hum Behav.

[24] Skivington K, Matthews L, Simpson SA, Craig P, Baird J, Blazeby JM (2021). A new framework for developing and evaluating complex interventions: update of Medical Research Council guidance. BMJ.

[25] O’ Donoghue M, Boland P, Leahy S, Galvin R, McManus J, Lisiecka D, et al. Exploring the perspectives of key stakeholders on the design and delivery of a cognitive rehabilitation intervention for people post-stroke. PLoS ONE 2022;17(6):e0269961. 10.1371/journal.pone.0269961 PMC9202836 35709170

[26] World Health Organization. Infection prevention and control during health care when coronavirus disease (COVID-19) is suspected or confirmed: interim guidance, 12 July 2021. World Health Organization. 2021.

[27] Eldridge SM, Lancaster GA, Campbell MJ, Thabane L, Hopewell S, Coleman CL (2016). Defining feasibility and pilot studies in preparation for randomised controlled trials: development of a conceptual framework. PLoS ONE.

[28] Eldridge SM, Chan CL, Campbell MJ, Bond CM, Hopewell S, Thabane L (2016). CONSORT 2010 statement: extension to randomised pilot and feasibility trials. BMJ.

[29] Takenoshita S, Terada S, Yoshida H, Yamaguchi M, Yabe M, Imai N (2019). Validation of Addenbrooke’s cognitive examination III for detecting mild cognitive impairment and dementia in Japan. BMC Geriatr.

[30] Tickle-Degnen L (2013). Nuts and bolts of conducting feasibility studies. Am J Occup Ther.

[31] Greenhalgh T, Hinton L, Finlay T, Macfarlane A, Fahy N, Clyde B (2019). Frameworks for supporting patient and public involvement in research: systematic review and co-design pilot. Health Expect.

[32] Hayes H, Buckland S, Tarpey M. INVOLVE briefing notes for researchers: involving the public in NHS. Public Health Soc Care Res Eastleigh INVOLVE. 2012.

[33] O’Cathain A, Croot L, Duncan E, Rousseau N, Sworn K, Turner KM (2019). Guidance on how to develop complex interventions to improve health and healthcare. BMJ Open.

[34] Staley K, Elliott J, Stewart D, Wilson R (2021). Who should I involve in my research and why? Patients, carers or the public?. Res Involv Engagem.

[35] Law M, Baptiste S, McColl M, Opzoomer A, Polatajko H, Pollock N (1990). The Canadian occupational performance measure: an outcome measure for occupational therapy. Can J Occup Ther.

[36] Billinger SA, Arena R, Bernhardt J, Eng JJ, Franklin BA, Johnson CM (2014). Physical activity and exercise recommendations for stroke survivors: a statement for healthcare professionals from the American Heart Association/American Stroke Association. Stroke.

[37] O’Donoghue M, Leahy S, Boland P, Galvin R, McManus J, Hayes S. Rehabilitation of cognitive deficits poststroke: systematic review and meta-analysis of randomized controlled trials. Stroke. 2022;STROKEAHA-121. 10.1161/STROKEAHA.121.034218 35109684

[38] Bruno D, Schurmann VS (2019). Addenbrooke’s cognitive examination III in the diagnosis of dementia: a critical review. Neuropsychiatr Dis Treat.

[39] Ozyemisci-Taskiran O, Batur EB, Yuksel S, Cengiz M, Karatas GK (2019). Validity and reliability of fatigue severity scale in stroke. Top Stroke Rehabil.

[40] Williams LS, Weinberger M, Harris LE, Clark DO, Biller J (1999). Development of a stroke-specific quality of life scale. Stroke.

[41] Post MW, Boosman H, Van Zandvoort MM, Passier PE, Rinkel GJ, Visser-Meily JM (2011). Development and validation of a short version of the Stroke Specific Quality of Life Scale. J Neurol Neurosurg Psychiatry.

[42] Salinas J, Sprinkhuizen SM, Ackerson T, Bernhardt J, Davie C, George MG (2016). An international standard set of patient-centered outcome measures after stroke. Stroke.

[43] Katzan IL, Lapin B (2018). PROMIS GH (patient-reported outcomes measurement information system Global Health) scale in stroke: a validation study. Stroke.

[44] Katzan IL, Fan Y, Uchino K, Griffith SD (2016). The PROMIS physical function scale: a promising scale for use in patients with ischemic stroke. Neurology.

[45] Yang SY, Lin CY, Lee YC, Chang JH (2017). The Canadian occupational performance measure for patients with stroke: a systematic review. J Phys Ther Sci.

[46] Watts G (2020). COVID-19 and the digital divide in the UK. Lancet Digit Health.

[47] Zolnierek KBH, Dimatteo MR (2009). Physician communication and patient adherence to treatment: a meta-analysis. Med Care.

[48] Clare L, Kudlicka A, Oyebode JR, Jones RW, Bayer A, Leroi I (2019). Goal-oriented cognitive rehabilitation for early-stage Alzheimer’s and related dementias: the GREAT RCT. Health Technol Assess Winch Engl.

[49] Hwi-Young C, Ki-Tae K, Jin-Hwa J (2015). Effects of computer assisted cognitive rehabilitation on brain wave, memory and attention of stroke patients: a randomized control trial. J Phys Ther Sci.

[50] Yoo C, Yong MH, Chung J, Yang Y (2015). Effect of computerized cognitive rehabilitation program on cognitive function and activities of living in stroke patients. J Phys Ther Sci.

[51] Kongkasuwan R, Voraakhom K, Pisolayabutra P, Maneechai P, Boonin J, Kuptniratsaikul V (2016). Creative art therapy to enhance rehabilitation for stroke patients: a randomized controlled trial. Clin Rehabil.

[52] Bo W, Lei M, Tao S, Jie LT, Qian L, Lin FQ (2019). Effects of combined intervention of physical exercise and cognitive training on cognitive function in stroke survivors with vascular cognitive impairment: a randomized controlled trial. Clin Rehabil.

[53] Zedlitz AM, Rietveld TC, Geurts AC, Fasotti L (2012). Cognitive and graded activity training can alleviate persistent fatigue after stroke: a randomized, controlled trial. Stroke.

[54] Laver K, Walker M, Ward N. Telerehabilitation for stroke is here to stay. But at what cost? Neurorehabil Neural Repair 2022;15459683221100492. 10.1177/15459683221100492 35527716

[55] Moore GF, Audrey S, Barker M, Bond L, Bonell C, Hardeman W (2015). Process evaluation of complex interventions: Medical Research Council guidance. BMJ.

[56] Masterson-Algar P, Burton CR, Rycroft-Malone J (2016). Process evaluations in neurological rehabilitation: a mixed-evidence systematic review and recommendations for future research. BMJ Open.

[57] McEwen D, O’Neil J, Miron-Celis M, Brosseau L (2019). Content reporting in post-stroke therapeutic circuit-class exercise programs in randomized control trials. Top Stroke Rehabil.

[58] French C, Pinnock H, Forbes G, Skene I, Taylor SJC (2020). Process evaluation within pragmatic randomised controlled trials: what is it, why is it done, and can we find it?-a systematic review. Trials.

[59] Hoffmann TC, Walker MF (2015). TIDieR-ing up’the reporting of interventions in stroke research: the importance of knowing what is in the ‘black box. Int J Stroke.

[60] Elkins JS, Khatabi T, Fung L, Rootenberg J, Johnston SC (2006). Recruiting subjects for acute stroke trials: a meta-analysis. Stroke.

[61] McGill K, Sackley CM, Godwin J, McGarry J, Brady MC (2020). A systematic review of the efficiency of recruitment to stroke rehabilitation randomised controlled trials. Trials.

[62] Pindus DM, Mullis R, Lim L, Wellwood I, Rundell AV, Abd Aziz NA (2018). Stroke survivors’ and informal caregivers’ experiences of primary care and community healthcare services–a systematic review and meta-ethnography. PLoS ONE.

[63] Howes S, Stephenson A, Murphy P, Deutsch J, Stokes M, Pedlow K (2022). Factors influencing the delivery of telerehabilitation for stroke: a systematic review. Physiotherapy.

