# Supplementary material for: OptiCogs: feasibility of a multicomponent intervention to rehabilitate people with cognitive impairment post-stroke
Source: Pilot Feasibility Stud. 2023 Oct 18;9:178. doi: 10.1186/s40814-023-01300-7 (PMC10583340; doi:10.1186/s40814-023-01300-7)
Supplement: Supplementary file 1 — Additional file 1: Table S1. Intervention schedule (OptiCogs). [file 40814_2023_1300_MOESM1_ESM.docx]

**Supplementary Table I: Intervention schedule (OptiCogs)**

| **Week 0** | Pre-intervention Assessment | |
| --- | --- | --- |
| **Week 1** | **Check-in with participants**  What is OptiCogs?   - Rule setting and boundaries of the OptiCogs group   Getting to know each other:   - Patient stories - Why are you here? - What is cognitive rehabilitation?   **Cognitive rehabilitation: Goal-setting**  Discussion, led by OT, around goal-setting completed in Week 0 as per the COPM.  Examples of goals and how we will work to achieve same.  **Group-based cognitive education**:  **Principle:** Knowledge  **Theme:** “You and your brain”   - Participants encouraged and facilitated to communicate about their shared experiences of cognitive deficits post-stroke - Discussion on how stroke can impact cognitive functioning and commonly affected areas of cognition e.g. memory, attention, problem-solving, executive functions, information processing. - How we can prioritise tasks, the importance of goal-setting, breaking big goals into achievable smaller tasks   **Transference of today’s learning to the home environment:**  Feedback from participants on running of session  Introduction of activity diary with fatigue measure (to be done as home activity)  Home activity: use your activity diary to document your levels of fatigue this week | **Check-in with participants**   1. Aerobic   Aerobic activity target of 10 mins per week of walking (1 x 10 minutes)   1. Individualised exercise session delivered via telehealth   **Exercise content:**  Demonstration of exercise components  Introduction to exercise log  Education on moderate intensity aerobic exercise  Coaching/ education on appropriate form  Live session with Chartered Physiotherapist  Sit to stand, 10 reps x 1 set  Squat, 10 reps x 1 set  Standing hip abduction, 10 reps x 1 set  Standing hip extension, 10 reps x 1 set  Wall Sit, 10 seconds x 1 set  Knee extensions, 10 reps x 1 set  Heel raises, 10 reps x 1 set  Seated rows, 10 reps x 1 set  **Learning Objectives- At the end of this week, participants will be able to:**   - Understand the core aspects of the intervention - Perform exercises with good form and identify a suitable amount of exercises to be performed - Complete one other home exercise programme and correctly complete exercise log |
| **One-to-one session week 1** | **Review participant story and goals collaboratively agreed in Week 0 (pre-assessment)**  **What is goal based on i.e. attention, memory or executive function**  **Individualised approach to the following based on goal and cognitive deficit(s) identified in Week 0 (pre-assessment)**   1. Education on specific task 2. Process training: Conscious to automatic skill of same 3. Strategy Training – if problem cannot be overcome, but does not cause a functional problem, then compensatory techniques may work – internal (mental processes: mental retracing steps when you lose your keys), external (physical objects or devices) and environmental (used by other people when person has insufficient awareness to utilise strategies independently). The above is dependent on participant level of awareness, intellectual awareness stage, emergent awareness or anticipatory awareness. 4. Functional Activities Training: 5. Application of learning to everyday life 6. Use of real life activities to work on underlying cognitive skills e.g. specific type of attention, memory or executive skill |  |
| **Week 2** | **Check-in with participants**  Review of the week   - Review of activity diary, fatigue and satisfaction levels - Expectations for upcoming session, opportunity for questions.   **Cognitive rehabilitation: Fatigue Management**   - Energy effectiveness techniques: focus on maximising available energy, prioritisation of energy reserves, pacing of activities, challenging unhelpful thoughts - Sleep hygiene - Mindfulness and relaxation   **Group-based cognitive education**  Principle: Fatigue Management  Theme “   - Cognitive fatigue management – How has cognitive fatigue impacted participants in their daily activities/ work?   **Transference of today’s learning to the home environment:**   - Environmental modification in the home: setting up the environment to reduce demands on energy reserves e.g. organising their space, setting up automatic systems such as bill-paying systems, card writing systems - Discussion, led by OT, around modification of activities to reduce cognitive fatigue and physical fatigue | 1. Aerobic   Aerobic activity target of 20 mins per week of walking (1 x 20 minutes or 2 x 10 minutes)   1. Individualised exercise session delivered via telehealth   Live session with Chartered Physiotherapist  As per week 1 with progressions to each exercise based on performance as determined by physiotherapist  **Learning Objectives- At the end of this week, participants will be able to:**   - Understand the concept of progressive overload in accordance with exercise guidelines for people post-stroke - Be confident in the safe completion of the exercise programme with appropriate form - Give examples of activity modification as management strategies for fatigue |
| **Week 3** | **Check-in with participants**  Review of the week, expectations for upcoming session, opportunity for questions.  **Cognitive Rehabilitation: Attention**  Attention process training  Compensatory strategies  **Group-based cognitive education**  Collaborative goal setting  Theme: You and your goals  • How does the environment impact cognition?  • What can we change that might help?  • Examples include external memory strategies, using bullet points, managing distraction, seeking help, managing and prioritising your workload/household duties, exercise, impact of other factors on cognition  **Transference of today’s learning to the home environment:**  Environmental modification in the home: Object location pairing e.g. strategic placing of car keys beside door.  Encourage an organised environment wherein there is a specified place for items e.g. phone, keys, bag etc | 1. Aerobic   Aerobic activity target of 30 mins per week of walking (1 x 30 minutes or 2 x 15 minutes)   1. Individualised exercise session delivered via telehealth   Live session with Chartered Physiotherapist  Exercises as per week 2 above, with progression to 10 reps x 2 sets  **Learning Objectives- At the end of this week, participants will be able to:**   - Discuss the role of the home environment and how it could be modified to encourage being more physically active and reduce distraction around the home - Set a goal to modify one barrier in the home setting |
| **One-to-one Week 3** | - Check in with individual participant - Review goal and strategies set on week 0 (Pre-assessment) - Review of what worked/what did not work and why - Participant opinions on why/ why not - Review education component from week 1 if necessary - Adjust goal if not achieved/ not feasible to achieve within time-frame - Proceed to goal 2 if appropriate, completing the four approaches from week 1 (education, process training, strategy training, functional activities training) |  |
| **Week 4** | **Check-in with participants**  Review of the week, expectations for upcoming session, opportunity for questions. Feedback from first home-based session.  **Cognitive Rehabilitation: Memory**   - Direct process training e.g. targeting working memory, encoding, retrieval via pen and paper activities, apps, activity - Training on internal and external memory strategies to improve compensating abilities - Development of routines and habits to promote automation of behaviours that support memory - Provision of external support in the form of compensatory memory aids - Cueing- provision relevant cues to aid information retrieval e.g. face-name recall, number recall, story recall, photo recall, - Chunking- organisation of information into small, relevant chunks e.g. making grocery lists, weekly schedules   **Group-based cognitive education**  Principle: Support  Theme: Stronger together   - Person-therapeutic relationship - How HCPs can support - How other PpS, family/friends can support - Community-support groups   **Transference of today’s learning to the home environment:**  Education regarding the transference of learning to activities of daily living and the home-setting, through the guidance of OT | 1. Aerobic   Aerobic activity target of 40 mins per week of walking (1 x 40 minutes or 2 x 20 minutes)   1. Individualised exercise session delivered via telehealth   Live session with Chartered Physiotherapist  As per week 3  **Learning Objectives- At the end of this week, participants will be able to:**   - Understand the benefits of physical activity post-stroke in relation to benefits in cognitive function - Identify how cognitive deficits post-stroke could impact on physical activity - Discuss and problem-solve strategies to mitigate against potential barriers to physical activity as a results of cognitive deficits post-stroke |
| **Week 5** | **Check-in with participants**  Review of the week, expectations for upcoming session, opportunity for questions.  **Cognitive Rehabilitation: Executive Function Part 1**  **Group-based cognitive education**  Principle: Problem-solving  Theme: You, the problem-solver  • Education on neuroplasticity and how the brain can change/ adapt  •Problem-solving in the home  •Discussion and shared ideas on the impact of cognitive difficulties on activities of daily living  **Transference of today’s learning to the home environment:**  Discussion, led by OT, around potential personality changes post-stroke and impulsivity  Education around social cues, regulation of comments and emotions in social situations | 1. Aerobic   Aerobic activity target of 50 mins per week of walking (1 x 50 minutes or 2 x 25 minutes)   1. Individualised exercise session delivered via telehealth   Live session with Chartered Physiotherapist  Exercises as per week 4, with progression to 10 repetitions x 3 sets   1. Individualised balance/ co-ordination tasks   **Learning Objectives- At the end of this week, participants will be able to:**   - Problem-solve any potential barriers encountered with performing the home exercise programme - Problem-solve potential solutions that could be implemented to physical activity goals/ actions going forward through group discussion |
| **One-to-one Week 5** | - Check in with individual participant - Review goal and strategies set on week 3 - What worked/what did not work - Participant opinions on why/ why not - Review education from week 1 and week 3 individualised sessions if necessary/appropriate. - Adjust goal number 2 if not achieved - Proceed to goal number 3 if appropriate, completing the four approaches from week 1 (education, process training, strategy training, functional activities training) |  |
| **Week 6** | **Check-in with participants**  Review of the week, expectations for upcoming session, opportunity for questions.  **Cognitive Rehabilitation: Executive Function Part 2**  **Group-based cognitive education**  Principle: Reintegration into the community  Theme: Your goals and beyond  Review goals and strategies learned  Next steps   - Action planning - Signpost to groups and services - Debrief and summary | 1. Aerobic   Aerobic activity target of 60 mins per week of walking (1 x 60 minutes or 2 x 30 minutes)   1. Individualised exercise session delivered via telehealth   Live session with Chartered Physiotherapist  As per week 5  **Learning Objectives- At the end of this week, participants will be able to:**   - Implement coping strategies should setbacks occur with their exercise programme going forward - Set an individualised goal in relation to their physical activity |
| **Week 7 Post-intervention assessment** | **Post-intervention Assessment Week 7** | |
